# Views of Hospital Nurses and Nursing Students on Nursing Engagement—Bridging the Gap Through Communication Courses

**DOI:** 10.3389/fpsyg.2022.915147

**Published:** 2022-06-29

**Authors:** Qing Huang, Jack Pun

**Affiliations:** ^1^School of Foreign Languages, Huizhou University, Huizhou, China; ^2^Department of English, City University of Hong Kong, Kowloon, Hong Kong SAR, China

**Keywords:** needs analysis, communication strategies, engagement, nurses, nursing students

## Abstract

**Background:**

Communicative engagement plays a significant role for effective nurse–patient communication. In the existing college nursing communication training within and outside China, there is a difference between what students are taught and what they can apply in their clinical placements.

**Aims:**

Using needs analysis, this mixed-methods study explored potential gaps between frontline hospital nurses' and college nursing students' perceptions of nurse–patient communicative engagement, and collated a list of effective engagement strategies for pedagogy.

**Methods:**

Surveys and interviews were conducted with key stakeholders, including 16 hospital nurses and 60 nursing students. A new scale named Nursing Engagement with Patients Scale (NEPS) was developed and validated to explore stakeholders' views on nursing engagement.

**Results:**

Differences between the views of nurses and students on engagement were identified. While frontline nurses affirmed the importance of engaging with patients while providing nursing care, nursing students were unsure about the concept and role of engagement, and how to enact it. A list of communication strategies that promote engagement was culled from the interviews with the experienced nurses.

**Implications:**

These can be used to inform nursing communication courses to bridge the gap between what nursing students are currently taught and what they will need in the workplace.

## Introduction

Effective nurse–patient communication can be achieved in a number of ways, one of which is the nursing professional's engagement with patients during their provision of medical care. The engagement has been studied by a number of researchers in the field (Roe et al., 2012; Bright et al., 2018), but the following description distills the various strands that are part of its practice into a concise definition: Nursing engagement is a nurse's active or proactive efforts to interact with a patient through two-way, patient-centered, communication in order to ensure that the nurse is understood by the patient, and also the nurse can assist the patient in revealing any concerns they may have, and provide empathy and rapport with the patient. Engagement helps achieve “holistic nursing,” characterized by Candlin and Candlin (2014, p. 260) as follows: “*in caring for persons across the lifespan and from all cultural backgrounds—whether sick or healthy-appropriate and ethical care is based on individual need: physical, emotional/psychological, social and spiritual, where all such needs are ineluctably interconnected in sites of nursing engagement*.”

Similar to nurses in other countries, nurses in China pay attention to the needs of their patients and play a major role in health care by engaging with patients on various levels. Yam and Rossiter (2000) found that Chinese nurses valued interpersonal communication that included engagement behaviors, such as encouraging patients to share their personal stories, or eliciting patients' emotional needs or unexpressed concerns that could help with their overall treatments. Similarly, Dong et al. (2016) found that Chinese nurses emphasized on the importance of meeting each patient's spiritual needs, one of the aspects of engagement, to help patients cope with stress, and help nurses build empathy with them.

Although nurses recognize the importance of engaging with patients, many often focus more on providing physical comfort than emotional support (Pun et al., 2018). Dong et al. (2016) interviewed Chinese nurses involved in looking after patients with terminal cancer and found that they were focused mainly on their patients' physical comfort during their nursing communication. Similarly, Jiang et al. (2015) used a nurse–patient interaction caring scale to measure the caring attitudes and behaviors of 260 nurses in mainland China. They found that nurses were more competent at providing operative care, such as drug delivery and injections, than they were at delivering expressive care, such as showing empathy and offering positive rapport, to engage with patients. More specifically, nurses holding higher positions (i.e., senior or supervisor nurses) had higher competency scores for caring and humanistic behaviors (i.e., features of engagement), in comparison to nurses in lower positions (Jiang et al., 2015).

The reasons behind the neglect by nurses of issues, such as emotional, spiritual, or environmental concerns, could lie in the hectic health care environment, where the patients feel vulnerable and the staff is frequently stressed. Meanwhile, meeting patients' holistic needs, which means all of their needs, including physical, emotional, spiritual, and environmental needs, demands more time and advanced interpersonal engagement skills (i.e., communication skills that promote engagement; Bramhall, 2014). Thus, nurses should have high level engagement skills, and the best way to ensure this is to teach engagement strategies (i.e., communication strategies that promote engagement) to novice nurses and nursing students.

### Teaching Engagement in Nursing Communication

The spoken modality is the most important form to enact engagement because it is the speech that is the primary means by which nurses interact with patients in urgent and high-risk clinical settings (Pangaltsos, 2011). The scope of the current study thus focuses on nurses' spoken efforts of engagement. To engage with patients in a better way, nurses need to employ a variety of strategies that will help them connect with their patients, and these engagement strategies are best learned through nursing college communication courses, as nurses may not have the opportunity to learn them while on the job from their busy clinical supervisors.

Studies have shown that many existing communication courses outside and within China only teach nursing students a very limited set of engagement-related communication skills, such as techniques for establishing empathy with patients (Shao et al., 2018), understanding patients (Farrell et al., 2007), gathering information (Bosher and Smalkoski, 2002), or counseling and delivering information (Lin et al., 2013). No course was found to include all the aspects of full nursing engagement. For example, in a study by Bosher and Smalkoski (2002), training in information-gathering techniques helped nursing students elicit patients' concerns but did not equip them with the skills to address patients' emotional needs. Thus, a dedicated communication course with a concerted focus on spoken engagement skills is needed. Students would learn about specific strategies for each engagement function, and these strategies can be generalized to work across different language and cultural contexts.

In the conventional communication skills training for nursing students worldwide, many trainees have expressed frustration about discrepancies between the communication training in classroom settings and their actual communicative needs in clinical practice. While teachers emphasized general principles of communication (“Be nice, kind, and patient”), nursing students were more concerned with practical communication skills that can help them to perform effectively in the workplace (Corlett, 2000). This mismatch suggests a need to develop better-designed communication courses that can teach practical engagement skills and can be actualized in real-life clinical scenarios. These courses should, thus, ideally be informed by the views and experiences of frontline nurses regarding engagement in clinical settings to give trainee nurses a better picture of what they will encounter in their future workplaces and how they should actively engage with patients. The views of frontline nurses can be identified through a needs analysis, to determine the specific learning goals that need to be addressed in the course design (Woodrow, 2018). For example, needs analysis has been used to elicit the views of stakeholders, such as hospital managers, nurse supervisors, and senior nurses, to develop language advancement courses that train nurses in effective clinical communication skills (Bosher and Smalkoski, 2002). These studies showed that needs analysis is an approach that can be used to determine the target learners' specific workplace language needs. The goal of the needs analysis in the current study was to answer the following research questions:

(1) What are the current perceptions of nurses and students in China regarding engagement in nursing communication?
(2) What specific kinds of communication strategies do the two groups think can lead to better engagement?

## Materials and Methods

Using needs analysis, the present study leads to the first step toward a larger project on nursing communication course design focused on engagement. To explore the gaps between students' needs in target communicative situations and their current communicative ability (Woodrow, 2018), this study used the mixed methods approach. The approach “adopts both qualitative and quantitative methods that work in a complementary way” (Paltridge and Phakiti, 2015, p. 28), and thus contributes to comprehensively understanding stakeholders' views on engagement. This approach was done by interviews for the qualitative arm, and surveys for the quantitative arm, in the current study, with 16 hospital nurses and 60 college nursing students, over a period of 3 months, from August 2019 to February 2020 (7 months in total). Their perceptions of engagement were then contrasted, and the gaps between these two groups of participants can be used to design engagement-focused courses in nursing communication [as suggested in Hafner and Miller (2019) and Woodrow (2018)].

### Participants

Sixteen nurses (including one nurse supervisor) from seven provincial grade-3 first-class hospitals, which are categorized as the top-ranking provincial hospitals in Guangdong province, and 60 students from a government-funded nursing college in Guangdong, participated in the study voluntarily *via* convenience sampling. The student volunteers were randomly selected from the 2019 cohort (*n* = 650) using a computer software. The demographic characteristics of the participating nurses and students are presented in Tables 1, 2, respectively.

**Table 1:** Demographic characteristics of the participating hospital nurses (*n* = 16).

**Variables**	**Category**	***n***
Age	21–30	10
	31–40	4
	41–50	2
Gender	Female	12
	Male	4
Position	Nurse supervisor	1
	Senior nurse	5
	Junior nurse	10
Years of experience as a registered nurse	5–10	10
	11–15	3
	16–20	1
	21–25	2

**Table 2:** Demographic characteristics of the participating students (*n* = 60).

**Variables**	**Category**	***N***	**%**
Age	18	3	5
	19	15	25
	20	41	68
	21	1	2
Gender	Male	6	10
	Female	54	90
Major	Nursing	60	100
L1	Mandarin	60	100

### Data Collection

#### Interviews

Each interview was ~25 min and was conducted in the participants' first language (i.e., Mandarin) by the first author, an instructor in health communication from the involved college. All interviews were audio-recorded for further analysis. To avoid any potential problems from the power imbalance between the interviewer and the students, the participating students were anonymized by being allocated random identification numbers by helpers. Due to the COVID-19 pandemic, the student interviews were conducted online through WeChat group chats. As there was a large number of student participants, the students were interviewed using a focus group format (four to five per group) to discuss how they viewed engagement with patients. The interviews with the hospital nurses, on the other hand, were conducted one-on-one in a quiet office to elicit their opinions fully. To protect the nurses' privacy, all of them were given pseudonyms in the interview transcripts.

The nurses were asked about their views and reflections on engagement practices and the related communication strategies, based on their extensive clinical experience, and also about novice nurses' perceptions and practices of engagement in clinical settings. Since the participating nursing students had had limited clinical experience with patients (e.g., intern-training), they were interviewed with a focus on their understanding of nursing engagement. All the interview questions were adapted from Roe et al., 2012 research on clinicians' perceptions of engagement with patients and are given in Appendix 1. The interviews were conducted in Mandarin Chinese to ensure that the interviewees, who were all Mandarin speakers, could express their ideas clearly and fluently in their mother tongue. The interview questions were translated from the original English into Chinese by two independent translators. The two versions were compared, analyzed, and modified until a consensus was reached. To further ensure content validity, five hospital nurses and five nursing students (all not part of the research) were recruited in a pilot study to help improve the clarity of the wording of the questions.

#### Questionnaires

Given that student participants outnumbered nurse participants, the former completed an anonymous survey, Nursing Engagement with Patients Scale (NEPS), before the interview, for gathering as many students' perceptions of engagement as possible. NEPS included three themes: students' attitudes toward learning engagement skills, perceived importance of engagement, and perceived ability to execute engagement. Three corresponding established scales that had been tested for validity and reliability in previous studies were adapted for this study (Coates, 1997; Langille et al., 2001; Rees et al., 2002). They had been successfully and simultaneously used in previous studies to gain insights into nursing students' communication skills (e.g., Abdrbo, 2017). Since these three established scales were used to explore the perceptions of interpersonal communication, the adapted items for this research narrowed their scope down to one aspect of interpersonal communication: engagement. Thus, the definition and explanation of engagement, as mentioned earlier, were highlighted in the adapted items. Appendix 2 shows the full version of the questionnaire used in this study. This new scale is named NEPS.

NEPS consists of 16 items, eight of which are written in the form of positive statements about perceptions of engagement while the other eight were negative statements. The inclusion of negatively worded items is an approach commonly employed to lessen the acquiescent response bias in a questionnaire (Qasem and Gul, 2014). These 16 items concern three themes and are deliberately mixed up in the scale so that similar items are not consecutive. Each theme covers roughly one-third of the items. Items 1, 4, 5, 10, 12, and 16 concern views on the learning of engagement skills, Items 3, 7, 8, 9, and 14 deal with the perceived importance of engagement, and Items 2, 6, 11, 13, and 15 involve perceived ability to execute engagement. Table 3 shows a summary of questionnaire items in NEPS.

**Table 3:** Summary of questionnaire items in NEPS.

**Items**	**Type**	**Description/theme**	**Example**
1, 4, 5, 10, 12, and 16	Opinion survey	Views on the learning of engagement skills	*In order to be a good nurse I must have good engagement skills (Item 1)*.
3, 7, 8, 9, and 14	Opinion survey	Perceived importance of engagement	*Checking for patient understanding is generally unnecessary (Item 3)*.
2, 6, 11, 13, and 15	Self-rating	Perceived ability to execute engagement	*I find it difficult to express empathy with patients (Item 2)*.

Responses to the statements in the questionnaire were on a Likert 6-point scoring scale (*Strongly agree, Agree, Neutral, Disagree, Strongly disagree*, and *I don't know*). To develop content validity and improve the clarity of the wording, each item on this scale was checked and slightly modified through several rounds of the panel discussion and pilot testing with two hospital nurses, two communication teachers, and two college nursing students (all not part of the research). The pilot test established that the amount of time needed to complete the survey was reasonable. The content validity and reliability of the scale were established using the Content Validity Index (CVI) developed by Waltz and Bausell (1983). The participants in the pilot testing were asked to rate each item based on relevance, clarity, simplicity, and ambiguity on a 4-point scale (see Table 4). All the items in NEPS had a CVI score over 0.75, indicating minimal potential threats (e.g., confounding variables, selection bias) to the construct validity of the instrument.

**Table 4:** Criteria for measuring content validity (Waltz and Bausell, 1983).

**1. Relevance**
1 = Not relevant
2 = Item needs some revision
3 = Clear but needs minor revision
4 = Very relevant
**2. Clarity**
1 = Not clear
2 = Item needs some revision
3 = Clear but needs minor revision
4 = Very clear
**3. Simplicity**
1= Not simple
2 = Item needs some revision
3 = Simple but needs minor revision
4 = Very simple
**4. Ambiguity**
1 = Ambiguous
2 = Item needs some revision
3 = Not ambiguous, but needs minor revision
4 = Meaning is clear

In addition, to confirm the factor structure underpinning the questions for each of the three themes of NEPS, corresponding to the three scales we adapted them from, we conducted factor analyses on our survey data. Our factor analyses confirmed the construct validity of the three themes: each theme had only one underlying factor. The KMO value for each theme was above 0.7, and the cumulative percentage of variance for each theme was above 50%. The three themes were actually already validated through factor analysis in the studies we adapted the questions from. Our study provides important confirmation of these earlier factor analyses. We also generated Cronbach's alphas for the three themes to measure their internal consistency (or reliability), since multiple Likert questions were used in each scale/theme. The results were all above the 0.7 thresholds for reliability for the questions on Theme 1, 2, and 3, respectively.

NEPS was distributed to students online through WeChat. The survey was self-administered, with brief written instructions for its completion. Before the survey, the teacher read the definition and the explanation of engagement for the participating students in the WeChat groups in the students' first language (i.e., Mandarin), in order to ensure they understood the concept of engagement. It took 15 min for students to complete the surveys each time through wjx (www.wjx.cn) using their mobile phones. It is an online software for designing questionnaires, administering them, and analyzing the results. Since all the questionnaire items were compulsory, it was set by wjx that the questionnaire could not be submitted unless all the items were answered. Thus, there were no missing data points for the questionnaires in this project. In order to gather more valid information, all the items on the questionnaires were written in the mother language of students (i.e., Chinese). The translation process was the same as that for the interview questions, as described earlier.

### Data Analysis

The questionnaires and interviews were subjected to quantitative and qualitative analyses, respectively. For the quantitative analysis of the questionnaires filled in by nursing students, using the statistical package SPSS, descriptive statistics were generated, such as the percentage of responses for each option for each item. The interviews were transcribed and input into *NVivo*, version 12, for qualitative analysis. The content of the transcripts was checked for accuracy by the interviewees together with the first author. Following the approach proposed by Braun and Clarke (2006), a thematic analysis of the transcripts was conducted. Themes regarding engagement were identified by re-reading the coded nodes and classifying sub-themes into broader patterns of meaning. A number of communication strategies promoting engagement practices were also identified in the data. To ensure the reliability of the coding framework, the interview data were independently coded by the first author and the second author according to a coding sheet. The two raters established inter-rater reliability of *k* > 0.8 using Cohen's Kappa coefficient.

### Ethics

The study was approved by the ethics committee of the involved nursing college before the study began. Consent forms were distributed by the first author to and signed by all participants before data collection that clearly explained the research aims, the confidentiality of the data, how the data would be used, and the participant's right to withdraw their participation for any reason.

## Results

The qualitative analyses showed that nurses and students had markedly different views about engagement. The divergences were identified in the following three major areas: (1) the role of engagement in patient care; (2) ways of enacting or realizing engagement; (3) what engagement strategies actually are. The students' perceptions were further supported by the results obtained through statistical analysis of their survey.

### The Role of Engagement in Patient Care

The nurse participants stated that engagement helped them improve patients' understanding, elicit patients' concerns and establish empathy and rapport with patients. For example, an experienced nurse named Lily (pseudonyms are used for all participants) shared her experience where her spoken engagement encouraged the patient to express their feelings and concerns and thus feel more relieved:

*Once a patient with a tumor asked me, ‘I am in so much pain. Am I dying soon?', after his body check report showed he was in poor health. I responded, ‘Why do you think so?', to encourage him to vent his feelings. The patient then told me how sad and in pain he was. Then, I said, ‘We have many patients who are in pain like you. We will help you. Do you remember when you first came here? Aren't you feeling better now?'... He calmed down after venting and said, ‘I feel better now. Thank you! I have to be brave.'* (Lily, female, 5 years of working experience).

Providing patients an opportunity or a space to express their concerns or vent their feelings is therefore one strategy for engaging with patients. Lily first recognized the patient's distress and then empathetically provided him an opportunity to express himself by asking an open-ended question, “*Why do you think so?*” The patient then told her more about his feelings. Thus, the patient's concerns were successfully elicited by the nurse and his emotional state was explored. Further, to communicate her empathy effectively, Lily used “*a lot of patients*” to help the patient feel less isolated, and “we” to suggest that there was a community of nurses all working together for the good of patients (“*We have a lot of patients who are in pain like you*”). By communicating empathy, Lily had communicated her understanding of the patient's feelings and expressed her willingness to support the patient, even reassuring him with empathetic, rapport-building responses, such as “*We will help you*” and “*Do you remember when you first came here? Aren't you feeling better now?*” These expressions are manifestations of strategies of engagement that effective nurses use to help patients feel that they are not alone and to allow nurses to establish empathy and connection with patients.

A similar example was given by a nurse supervisor, Mary, to illustrate her belief that engagement can help nurses meet patients' emotional needs and increase patients' cooperation with clinical treatments:

*I once gave an IV infusion to a patient and incidentally found that the wound on her right ankle was infected. I asked if she was in pain, and she suddenly cried and said, ‘I have been here for a few days. No one asked me!'. I said, ‘Don't worry! I will ask the doctor to put another dressing on your wound now'... In the following days, she trusted me a lot and cooperated with me during her treatments* (Mary, female, 24 years of working experience).

In this case, the patient was in pain not because of her ankle but because of the loneliness of having no one to talk with about her condition. Because the nurse engaged with the patient by asking if she was in pain, the patient felt cared for and respected, as evidenced by her subsequent cooperation with the nurse. The nurse supervisor further communicated empathetic engagement by offering reassurance (“*Don't worry! I will ask the doctor to put another dressing on your wound now*”). Despite the brevity of this spoken interaction, Mary was able to use engagement strategies to elicit the patient's concerns and establish empathy, rapport, and mutual trust, and thus perhaps better clinical outcomes.

In contrast, some nursing students did not share this view of the importance of engagement. Based on their survey results, some students held negative attitudes toward the importance of engagement (Theme 1 in NEPS) and learning engagement skills (Theme 3 in NEPS). Tables 5, 6 show the percentages of each response for each item in Theme 1 and Theme 3, respectively. As can be seen from Table 5, some students did not see the value of engagement with patients but instead would rather focus on learning procedural knowledge about clinical tasks. For example, 18.33% of the students agreed that acknowledging the patient's experience was not necessary for nurse–patient relationships (Item 14), which is a way to promote engagement. As one participating student said,

**Table 5:** Percentages of each response for each item on perceived importance of engagement (Theme 1).

**Item**	**Attitude**	***n***	**%**
Item 3: Checking for patient understanding is generally unnecessary*	I don't know	0	0%
	Strongly disagree	6	10%
	Disagree	30	50%
	Neutral	17	28%
	Agree	7	12%
	Strongly agree	0	0%
Item 7: Dealing with the emotional problems of patients is the responsibility of psychiatrists, psychologists and social workers, not nurses*	I don't know	1	1.67%
	Strongly disagree	4	6.67%
	Disagree	41	68.33%
	Neutral	10	16.67%
	Agree	4	6.67%
	Strongly agree	0	0%
Item 8: Good nurse–patient communication improves patients' health outcomes	I don't know	0	0%
	Strongly disagree	1	1.67%
	Disagree	1	1.67%
	Neutral	17	28.33%
	Agree	38	63.33%
	Strongly agree	3	5%
Item 9: Addressing patients' emotions and psychosocial issues is absolutely essential in health-solving today	I don't know	0	0%
	Strongly disagree	0	0%
	Disagree	0	0%
	Neutral	18	30%
	Agree	41	68.33%
	Strongly agree	1	1.67%
Item 14: Acknowledging the patient's experience is not necessary in nurse-patient relationships*	I don't know	2	3.33%
	Strongly disagree	5	8.33%
	Disagree	28	46.67%
	Neutral	14	23.33%
	Agree	11	18.33%
	Strongly agree	0	0

^*^Negative statement.

**Table 6:** Percentages of each response for each item on views on the learning of engagement skills (Theme 3).

**Item**	**Attitude**	***n***	**%**
Item 1: In order to be a good nurse I must have good engagement skills	I don't know	0	0%
	Strongly disagree	4	6.67%
	Disagree	0	0%
	Neutral	6	10%
	Agree	29	48.33%
	Strongly agree	21	35%
Item 4: Developing my engagement skills is just as important as developing my knowledge of nursing	I don't know	0	0%
	Strongly disagree	0	0%
	Disagree	0	0%
	Neutral	11	18.33%
	Agree	37	61.67%
	Strongly agree	12	20%
Item 5: Learning engagement skills has helped or will help me understand patients	I don't know	0	0%
	Strongly disagree	0	0%
	Disagree	0	0%
	Neutral	5	8.33%
	Agree	47	78.33%
	Strongly agree	8	13.33%
Item 10: Learning engagement skills has improved or will improve my ability to communicate with patients	I don't know	0	0%
	Strongly disagree	1	1.67%
	Disagree	0	0%
	Neutral	6	10%
	Agree	46	76.67%
	Strongly agree	7	11.67%
Item 12: Learning engagement skills is fun	I don't know	2	3.33%
	Strongly disagree	1	1.67%
	Disagree	3	5%
	Neutral	26	43.33%
	Agree	28	46.67%
	Strongly agree	0	0%
Item 16: Learning engagement skills has not helped or will not help me elicit patients' concerns*	I don't know	2	3.33%
	Strongly disagree	3	5%
	Disagree	34	56.67%
	Neutral	9	15%
	Agree	12	20%
	Strongly agree	0	0

^*^Negative statement.

*Compared with communication skills related to promoting engagement, it is much more important for us to focus on learning procedural knowledge of the different clinical tasks we would have to perform* (Student 02).

Another example is related to the concept of small talk, an informal type of conversation. It can be about everyday life unrelated to the medical conditions of the patients (Bosher and Smalkoski, 2002), such as “*What are you going to have for dinner?*” Some students thought it was unnecessary for them to have small talk with patients during nursing procedures. One student said,

*I don't think it is necessary to chat with patients during nursing procedures. It will interfere with my nursing work, and possibly arouse the dissatisfaction of the patients because they may think I am nosy* (Student 31).

Unlike the students, many nurse participants believed that having small talk with patients was an important way to establish closer and more trusting nurse-patient relationships, relieve anxiety, assist in the healing process, and thus achieve better clinical outcomes. Frontline health care workers in Hong Kong, in a study by Slade et al. (2015), had a similar view, saying that chatting with patients about aspects of daily life can put patients at ease and reduce the professional distance between nurses and patients.

One salient fact we can derive from the tables is a considerable proportion of students held positive attitudes toward the value of engagement in patient care (Table 5) and positive opinions on learning engagement skills (Table 6). However, among the students who acknowledged the importance of engagement, the attitudes of nearly all of them were “*agree*” rather than “*strongly agree*.” For example, only one student (1.67%) strongly agreed that addressing patients' emotions and psychosocial issues was absolutely essential in health-solving today (Item 9). This is possible because they were not clear about what was important about engagement. In the subsequent interviews, it was hard for quite a few students who valued engagement to explain why they thought it was important to engage with the patients during nursing care. As one participating student said,

*My first feeling is that engagement is important, because that's what everyone says, but when asked why, I couldn't explain it* (Student 01).

Similarly, among the students who held positive attitudes toward learning engagement, the responses of the majority were “*agree*” while a few were “*strongly agree*”, suggesting that their degree of positiveness was not strong. For example, only three students (5%) strongly disagreed that learning engagement skills had not helped or would not help them elicit patients' concerns (Item 16). The reason may be that students did not really know what engagement was and why they had to learn it. They chose positive responses only by intuition or common sense (i.e., what they thought were the expectations of society). As one student said in the interview,

*It is important to learn how to engage with the patients is a clichéd view for me. It is not that important to me* (Student 42).

### Ways of Enacting Engagement

Another difference between nurses and students is that many students lacked confidence in enacting engagement as they did not know how to enact engagement (i.e., what strategies to use, or what to say) even when they were willing to do so. It was supported by statistical results generated from the questionnaire. Table 7 displays detailed data on students' perceived ability to execute engagement (Theme 2 in NEPS), with the percentages of responses for each item. This theme was included to gauge the nursing students' self-perceived ability to execute engagement with patients. Based on Table 7, the majority of students were not confident in their ability to engage communicatively with patients. For example, more than half of the students (51.67%) lacked confidence in their ability to talk to patients from different backgrounds, and only two of the students (3.31%) thought they could do a good job in this respect (Item 6). Many students further expressed their worries in the interviews:

**Table 7:** Percentages of each response for each item on perceived ability to execute engagement (Theme 2).

**Item**	**Attitude**	***n***	**%**
Item 2: I find it difficult to express empathy with patients*	I don't know	0	0%
	Strongly disagree	0	0%
	Disagree	7	11.67%
	Neutral	25	41.67%
	Agree	24	40%
	Strongly agree	4	6.67%
Item 6: I lack confidence in my ability to talk to patients from backgrounds different from my own*	I don't know	3	5%
	Strongly disagree	0	0%
	Disagree	2	3.33%
	Neutral	24	40%
	Agree	27	45%
	Strongly agree	4	6.67%
Item 11: I find it hard to get my point of view across to patients*	I don't know	3	5%
	Strongly disagree	0	0%
	Disagree	2	3.33%
	Neutral	14	23.33%
	Agree	32	53.33%
	Strongly agree	9	15%
Item 13: While communicating with a patient, I am able to verbalize to the patient that I comprehend what is being said	I don't know	7	11.67%
	Strongly disagree	1	1.67%
	Disagree	6	10%
	Neutral	22	36.67%
	Agree	23	38.33%
	Strongly agree	1	1.67%
Item 15: I find it hard to elicit patients' needs and thoughts*	I don't know	6	10%
	Strongly disagree	1	1.67%
	Disagree	3	5%
	Neutral	26	43.33%
	Agree	22	36.67%
	Strongly agree	2	3.33%

^*^Negative statement.

*What worries me most is that the patient can't understand or misunderstands what I'm saying* (Student 34).

*I know it is important to engage with patients, but I don't know how to* (Student 03).

*I will be most embarrassed to meet patients who don't like to talk. If I ask and then the patient simply answers the questions without adding much information, it is too awkward* (Student 05).

When confronted with possible chances of enacting engagement, students and nurses expressed completely different ways of dealing with it. For example, many student interviewees said that they put minimal effort into responding to patients' questions, especially when they did not have an answer. As one of the students noted,

*I am afraid that I will give patients the wrong information and mislead them* (Student 02).

Unlike the students, nurses believed that cursory responses annoy patients and asserted that it was always important to engage with patients by actively addressing their questions even if there was no immediate explanation or answer to give (e.g., nurses could offer to find someone who had the answer). Frontline nurses believed that engagement strategies could go a long way toward addressing patients' emotional needs in such situations, making patients feel heard and respected.

Another difference is that many students felt that they could rely on basic medical terminology to communicate with patients, believing that using medical terms was equivalent to providing patient care. Nurses, by contrast, said that they always aimed to maximize patient comprehension, avoiding using jargon on purpose, as an engagement strategy to ensure the patient's comprehension. More examples of engagement strategies will be presented in the next section.

### What Engagement Strategies Actually Are

The nurses believed that they could engage better with patients through the use of various engagement strategies, and had a clear understanding of what engagement strategies were, as illustrated in the examples given earlier. On the contrary, when nursing students were asked about engagement strategies, the majority did not really understand the concept and interpreted the question as being about etiquette, or how they should behave when talking with patients. This was clear from their responses in the interviews: they could only suggest being polite or kind to patients when interacting with them. Unlike experienced nurses, they did not know of any specific communication strategies they needed to use to achieve the functions of engagement. This is probably because they had not received any training on this issue.

The engagement strategies identified from the interviews with the experienced nurses (listed in Table 8) can be divided into three categories corresponding to the three types or functions of engagement included in the definition of engagement: improving patients' understanding, eliciting patients' concerns, and establishing empathy and rapport. These will be discussed in the following subsections:

**Table 8:** Engagement strategies identified from nurses' interviews.

**Types of engagement**	**Engagement strategies**
Category 1: Improving patients' understanding	(1) Ensuring that offered information is easily comprehended (2) Avoiding jargon or spontaneously explaining it well
Category 2: Eliciting patients' concerns	(1) Asking open-ended questions (2) Asking focused questions (3) Exploring personal health-related habits or asking questions about sensitive topics (4) Employing probes
Category 3: Establishing empathy and rapport with patients	(1) Recognizing or creating the opportunity to show empathy when necessary (2) Exploring the patient's emotional state (3) Stating an intention to help and support (4) Expressing understanding of a patient's feelings or situation (5) Showing respect or expressing praise for a patient (6) Paying attention and responding to patient's spoken or non-spoken cues that can manifest their feelings (7) Offering empathetic or supportive comments (8) Offering spoken reassurance through which a sense of hope is created (9) Making small talk with the patients about some unimportant subjects during the procedures (10) Assisting patients to connect with their significant others (11) Responding actively to questions raised by the patients, even if the nurse does not know the answers

#### Improving Patients' Understanding

The nurse interviewees noted that both nurses' understanding of patients and patients' understanding of nurses were essential to achieving engagement. This two-way understanding was recognized in the definition of “*engagement*” given earlier. Strategies to improve patients' understanding mentioned by the nurses included offering information that is easily comprehended and avoiding jargon. One nurse shared the following:

*Patients always ask, ‘What medicine is it?'. If I tell them the exact names of the drugs, they wouldn't understand them because I would be using medical jargon... Patients are likely to feel nervous, as such drug names are sometimes very long and complicated. This may make them feel they are seriously ill. We can just tell them the functions of the medicine with everyday expressions. For example, we can say, ‘It is good for your lungs' or ‘It can stop vomiting'* (Connie, female, 30 years of working experience).

With the communication strategies mentioned above, simplifying medical information and using a layman's language without leaving out any essential information, nurses can better engage with patients and thus can ensure better patient compliance and outcomes.

#### Eliciting Patients' Concerns

The most common communication strategies nurses mentioned that they had used to elicit patients' concerns were asking open-ended questions (to allow patients free expression), asking focused questions (to limit the range of the response), employing probes (to gather further detailed information on a specific topic), and exploring personal health-related habits or asking questions about sensitive topics. Nurse Nana said,

*Sometimes, patients can't express themselves clearly and just say they are uncomfortable. Then, we need to ask for further details focusing on the specific area where the patient didn't feel well by asking focused questions or using probes* (Nana, female, 19 years of working experience).

The nurse supervisor also gave an example of asking open-ended questions:

*When I want to find out about patients' sleep quality, I will ask, ‘How did you sleep last night?', in order to encourage patients to say more, rather than a yes-no question, ‘Did you sleep well last night?'* (Mary, female, 24 years of working experience).

#### Establishing Empathy and Rapport With Patients

The strategies applied by the nurses mentioned for establishing empathy and rapport with patients included: paying attention and responding to patients' cues that could indicate their feelings, expressing understanding of a patient's feelings or situation, and offering empathetic or supportive comments, especially after learning about a patient's unpleasant or even miserable experiences, praising patients, and offering spoken reassurance. As the nurse supervisor described,

*My patient once told me she had three miscarriages, and this was her fifth pregnancy. I said, ‘Oh, it's really hard on you.'. During the fetal heart monitoring, I asked her to feel baby's heartbeat and said, ‘Listen, the baby's heartbeat is so powerful. The baby can feel that you are a great mom!'* (Mary, female, 24 years of working experience).

Through the nurse supervisor's empathetic feedback (“*Oh, it's really hard on you*.”) and praise for the patient (“…*you are a great mom!*”), the patient would have felt understood and supported. Another similar example was provided by nurse Apple, whose patient's tension was relieved after he was told that he was not the only person to have had blood in the urine after an operation:

*One of my patients told my nervously, ‘I have blood in my urine,' and I reassured him by saying, ‘Don't worry. It is normal because you've just had an operation'* (Apple, female, 13 years of working experience).

The above two examples clearly illustrate that paying attention to patients' emotional states is an important part of a nurse's job. Both nurses interpreted medical results positively for the patients, adding empathetic and supportive comments to reassure patients and establish empathy with them.

### New Engagement Strategies

Most of the communication strategies mentioned by the nurse participants, such as ensuring that information offered are easily comprehended (Van Zanten et al., 2007; Slade et al., 2015), can be found in the literature. However, several new communication strategies were suggested by the nurse participants in this study. First, they suggested the strategy of assisting patients to connect with their family members and friends. One nurse described how she helped an elderly patient adhere to the clinical treatments with this strategy:

*The patient refused to take medicine after the surgery and wanted to go home immediately... because he missed his wife, who was too old to walk here to see him... I encouraged him to have a video chat with his wife... He finally agreed to stay in the hospital* (Mary, female, 24 years of working experience).

By facilitating the video chat with his wife, the patient's emotional needs were addressed, and the nurse probably provided him relief from his anxiety, depression, and pain. The second new strategy identified is that of actively responding to questions raised by patients, even if the nurse does not know the answers. While many strategies identified in previous studies are aimed at eliciting patients' responses (Van Zanten et al., 2007; Slade et al., 2015), this strategy is about ensuring that nurses' responses make patients feel heard and respected. Nurses should offer to find answers to questions raised by patients even if they are not able to immediately answer them, to reassure patients that the matter will be handled. As a nurse said,

*Even if I don't know the answers, I will actively respond to the patients. I can say, ‘I am not very sure about this. I will ask the doctor and come back to you later'* (Lily, female, 5 years of working experience).

Although these communication strategies were not among those reported in the previous literature on nursing communication, our findings suggest that they can lead to positive nursing engagement.

## Discussion

The qualitative findings show that the paramount importance of engagement was recognized by all the interviewed hospital nurses. They stated that engagement with patients meant going beyond attending to patients' immediate symptoms or pain during nursing care. This attention to the various dimensions of patients' needs is at the core of engagement, and the nurses' reflections on their behaviors showcased typical engagement behaviors as defined in the previous literature (Roe et al., 2012; Bright et al., 2018). The analysis of frontline nurses' views in this current study established the following three main functions of nursing engagement: (1) to improve patients' understanding of what the nurse is saying, especially in relation to the patients' medical conditions, treatments, and procedures; (2) to elicit patients' concerns, through the use of communication strategies that encourage patients to express themselves more in terms of their different needs (e.g., physical, emotional, spiritual, and environmental concerns); and (3) to establish empathy and rapport with patients, through communication strategies that convey to patients that they are understood, their fears and concerns heard, and that their value as people is recognized.

In contrast to the views of experienced nurses, the nursing students, based on their qualitative and quantitative findings above, tended to concentrate on learning procedural knowledge of different clinical tasks and did not fully see the value of engagement, nor understand how to enact engagement with patients. With regard to communication training, the divergent views between nurses and students highlight their different priorities. Frontline nurses see communication skills as key to providing comprehensive care to patients because of how effective communication strategies can be at enacting engagement. Nursing students, however, pay more attention to the rote learning of medical terminology, which they believe is how they can be better nurses. The nurse supervisor interviewed for this study said that this mistaken belief was common among students with limited clinical working experience, suggesting a gap in nursing communication education: students are not being taught the full range of communication skills necessary for successful engagement with patients. In traditional nursing education in China, for example, the focus is on teaching students to memorize medical concepts and jargon (Peng and Ran, 2009). Thus, there is a real need for nursing colleges to have focused communication courses that specifically teach nursing engagement skills. While training in medical procedures and terminology is important for nurses' collaborative work with doctors, training in the use of communicative engagement strategies is also essential for the achievement of effective patient care, and the two strands can be combined in redesigned nursing communication courses. The frontline nurses interviewed for this study expressed a wish for nursing students to learn engagement strategies as part of their training at nursing college before they enter the workplace, and strongly suggested that the course materials should involve the study of samples of authentic nurse-patient hospital interactions so that students can learn how to use communication strategies to engage with patients while simultaneously carrying out the necessary medical procedures and other nursing tasks.

The strategies discussed in this current study are not specific to China but are transferrable to other contexts and regions worldwide. As mentioned earlier, the majority of the strategies are consistent with those from previous studies, while the new strategies are also universally relevant. For example, the strategy of assisting patients to connect with their significant others is consistent with the Roy adaptation model (Roy, 2009, 2011), which nurses all around the world use to ensure holistic care, in which the relationships with others who are meaningful to patients play a crucial role.

## Conclusion

This study is one of the first to explore Chinese nurses' and nursing student's perceptions of engagement in nurse-patient communication. A new scale, NEPS, was thus developed and validated and this scale can also be applied to explore stakeholders' views on engagement in the future. Using surveys and interviews, the study explored frontline hospital nurses' and college nursing students' divergent views on engagement and suggested that these gaps should be addressed pedagogically in nursing college communication courses. While experienced nurses emphasized the importance of effectively engaging with patients through various communication strategies, the students showed little understanding of how to engage with patients, nor of the important role of engagement in nursing. There is a gap between what nursing students are taught in existing nursing communication training, and what they need to do in their clinical placements. Given proper training and practice in the relevant communication skills, nursing students will be better equipped to engage effectively with patients once they graduate, in spite of the time constraints and stressful conditions that sometimes make being patient-centered quite challenging. Engaging with patients does not necessarily require a lot of time, and if nurses are well-trained, they will be able to use the necessary communication strategies automatically, even under stressful conditions, and thus be more effective as nurses.

The results have pedagogical and clinical implications for nurses and nurse educators everywhere. Nurse participants in this study thought it was important to provide students with authentic, contextualized samples of nurse–patient interactions collected from hospitals—a broad range of interactional nursing scenarios that students can learn effective engagement practices. Such a needs-aligned course will address the current gaps between what students learn and what they can use in clinical settings. It is the job of communication instructors at nursing colleges to teach these workplace-relevant skills, which can meet nursing students' occupational needs. The communication strategies identified in this study, embedded within a well-designed course that focuses on how they function to realize engagement, as exemplified by authentic interactional data, will help novice nurses quickly get up to speed once they are in the workplace and thus provide better nursing care, and, in turn, improved clinical outcomes. In terms of future directions extending from the current research, it is worth exploring whether more statistical methods can provide better insights concerning teaching engagement in nursing communication.

## Data Availability Statement

The original contributions presented in the study are included in the article/Supplementary Material, further inquiries can be directed to the corresponding author/s.

## Ethics Statement

The studies involving human participants were reviewed and approved by Huizhou Health Sciences Polytechnic and City University of Hong Kong. The patients/participants provided their written informed consent to participate in this study.

## Author Contributions

QH and JP designed the study and wrote the manuscript. QH was responsible for data collection, data analysis, and drafting of the manuscript. JP revised and edited this manuscript. Both authors contributed to the article and approved the submitted version.

## Conflict of Interest

The authors declare that the research was conducted in the absence of any commercial or financial relationships that could be construed as a potential conflict of interest.

## Publisher's Note

All claims expressed in this article are solely those of the authors and do not necessarily represent those of their affiliated organizations, or those of the publisher, the editors and the reviewers. Any product that may be evaluated in this article, or claim that may be made by its manufacturer, is not guaranteed or endorsed by the publisher.
