# Supplementary material for: Views of Hospital Nurses and Nursing Students on Nursing Engagement—Bridging the Gap Through Communication Courses
Source: Front Psychol. 2022 Jun 29;13:915147. doi: 10.3389/fpsyg.2022.915147 (PMC9277507; doi:10.3389/fpsyg.2022.915147)
Supplement: Supplementary file 1 [file Table_1.docx]

# Appendices

**Appendix 1.** Interview questions

**i. Interview questions for hospital nurses**

1. How long have you been employed as a registered nurse?

2. How many non-Chinese patients do you usually serve every week?

3. When I say the term “nursing engagement with patient” what things come to your mind?

4. Do you think spoken language plays an important role in engaging with patients? What do you usually say to engage with your patients?

5. Can you provide some examples of good spoken engagement with patient based on your experiences?

6. Do you have any specific spoken strategy to better understand patients’ ideas?

7. What do you say to make yourself better understood by your patients?

8. What do you say to elicit as many patients’ concerns as possible, especially about their emotional needs?

9. How do you build empathy and rapport with patients? Any spoken strategy?

10. What similarities and differences should be paid attention to when you engage with Chinese patients and non-Chinese patients? What are possible reasons for the differences?

11. What do you think is the most difficult or problematic part for inexperienced nurses to do when engaging with the patients, especially through spoken communication?

12.What do you expect for nursing students to learn at college regarding spoken communication with patients?

**ii. Interview questions for nursing students**

1. Which class are you in?

2. Have you attended any training on communication skills before? If so, what was taught in the course?

3. When I say the term “nursing engagement with patient” what things come to your mind?

4. What is good spoken engagement with a patient? any example?

5. Do you think spoken language plays an important role in engaging with patients? Why?

6. What would you say to better understand patients’ ideas?

7. What would you say to make yourself better understood by your patients?

8. What would you say to elicit as many patients’ concerns as possible, especially about their emotional needs?

9. How would you build empathy and rapport with patients? Any spoken strategy?

10. What do you think would be the most difficult or problematic part when engaging with the patients (or learning how to engage with the patients), especially through spoken communication?

**Appendix 2.** Nursing Engagement with Patients Scale (NEPS)

Please read the following statements about nursing engagement with patients. Indicate whether you agree or disagree with all of the statements by circling the most appropriate response. Remember,

0 = I don’t know

1 = strongly disagree

2 = disagree

3 = neutral

4 = agree

5 = strongly agree

| 1. In order to be a good nurse I must have good engagement skills | 0 | 1 | 2 | 3 | 4 | 5 |
| --- | --- | --- | --- | --- | --- | --- |
| 2. I find it difficult to express empathy with patients | 0 | 1 | 2 | 3 | 4 | 5 |
| 3. Checking for patient understanding is generally unnecessary | 0 | 1 | 2 | 3 | 4 | 5 |
| 4. Developing my engagement skills is just as important as developing my knowledge of nursing | 0 | 1 | 2 | 3 | 4 | 5 |
| 5. Learning engagement skills has helped or will help me understand patients | 0 | 1 | 2 | 3 | 4 | 5 |
| 6. I lack confidence in my ability to talk to patients from backgrounds different from my own | 0 | 1 | 2 | 3 | 4 | 5 |
| 7. Dealing with the emotional problems of patients is the responsibility of psychiatrists, psychologists and social workers, not nurses | 0 | 1 | 2 | 3 | 4 | 5 |
| 8. Good nurse-patient communication improves patients' health outcomes | 0 | 1 | 2 | 3 | 4 | 5 |
| 9. Addressing patients' emotions and psychosocial issues is absolutely essential in nursing today | 0 | 1 | 2 | 3 | 4 | 5 |
| 10. Learning engagement skills has improved or will improve my ability to communicate with patients | 0 | 1 | 2 | 3 | 4 | 5 |
| 11. I find it hard to get my point of view across to patients | 0 | 1 | 2 | 3 | 4 | 5 |
| 12. Learning engagement skills is fun | 0 | 1 | 2 | 3 | 4 | 5 |
| 13. While communicating with a patient, I am able to verbalize to the patient that I comprehend what is being said | 0 | 1 | 2 | 3 | 4 | 5 |
| 14. Acknowledging the patient's experience is not necessary in nurse-patient relationships | 0 | 1 | 2 | 3 | 4 | 5 |
| 15. I find it hard to elicit needs and thoughts of patients from backgrounds different from my own | 0 | 1 | 2 | 3 | 4 | 5 |
| 16. Learning engagement skills has not helped or will not help me elicit patients' concerns | 0 | 1 | 2 | 3 | 4 | 5 |
